# Differences in Cellular Immune Competence Explain Parasitoid Resistance for Two Coleopteran Species

**DOI:** 10.1371/journal.pone.0108795

**Published:** 2014-09-26

**Authors:** Lisa Fors, Robert Markus, Ulrich Theopold, Peter A. Hambäck

**Affiliations:** 1 Department of Ecology, Environment and Plant Sciences, Stockholm University, Stockholm, Sweden; 2 Department of Neurosciences, Stockholm University, Stockholm, Sweden; 3 Department of Molecular Biosciences, The Wenner-Gren Institute, Stockholm University, Stockholm, Sweden; USDA-Agricultural Research Service, United States of America

## Abstract

The immune defence of an organism is evolving continuously, causing counteradaptations in interacting species, which in turn affect other ecological and evolutionary processes. Until recently comparative studies of species interactions and immunity, combining information from both ecological and immunological fields, have been rare. The cellular immune defense in insects, mainly mediated by circulating hemocytes, has been studied primarily in Lepidoptera and Diptera, whereas corresponding information about coleopteran species is still scarce. In the study presented here, we used two closely related chrysomelids, *Galerucella pusilla* and *G*. *calmariensis* (Coleoptera), both attacked by the same parasitoid, *Asecodes parviclava* (Hymenoptera). In order to investigate the structure of the immune system in *Galerucella* and to detect possible differences between the two species, we combined ecological studies with controlled parasitism experiments, followed by an investigation of the cell composition in the larval hemolymph. We found a striking difference in parasitism rate between the species, as well as in the level of successful immune response (i.e. encapsulation and melanisation of parasitoid eggs), with *G. pusilla* showing a much more potent immune defense than *G. calmariensis*. These differences were linked to differences in the larval cell composition, where hemocyte subsets in both naïve and parasitised individuals differed significantly between the species. In particular, the hemocytes shown to be active in the encapsulation process; phagocytes, lamellocytes and granulocytes, differ between the species, indicating that the cell composition reflects the ability to defend against the parasitoid.

## Introduction

The immune system of an organism provides protection against parasites, parasitoids and pathogens, thereby strongly influencing its fitness [1]. To adapt to novel threats, the immune defense of the host evolves continuously, causing counteradaptations in the intruder’s virulence. Both the innate and acquired immunity may be affected in these processes. As a consequence, even comparatively simple immune systems show considerable variation in the usage of effector mechanisms. The strength of the immune system and the intruder’s virulence will affect the strength of species interactions and thereby other ecological processes and evolutionary trajectories. For instance, geographic variation in the strength of species interactions and selection strengths may be a consequence both of changes in food web structure and because of geographic variation in immune competence [2]. Until recently, however, eco-evolutionary studies of species interactions have rarely included analyses of immune function [3], [4].

The immune system of insects consists of several defence mechanisms, including cellular and humoral responses. In humoral defenses soluble molecules, toxic to intruding parasites and pathogens, are secreted into the open circulatory system [5], [6], acting either directly on the invader or by altering the immune response of the insect [7]. Cellular defenses include several mechanisms that are directly mediated by immune cells (hemocytes), such as clotting, phagocytosis and encapsulation [8]–[10]. Clotting is the coagulation of hemolymph at a wound site in order to seal the wound, especially important in the open circulatory system of insects [11]. Phagocytosis is a process where intruding objects are ingested and destroyed by specialised hemocytes [12]. If the intruder is too large to be phagocytosed, the common immune response is instead encapsulation, a process where a capsule is formed around foreign objects (such as parasitoid eggs) by aggregating cells. The encapsulation process usually begins 4–6 hours after a parasitoid attack and is completed after approximately 48 hours [13]. The process is accompanied by the production of melanin, resulting in a localized blackening around the encapsulated object and of the tissue at the wound site [14]. Due to crosslinking of its protein compounds the capsule hardens, leading to the death of the enclosed intruder [15]–[17]. Cellular defenses have been studied mainly in Diptera [18] and Lepidoptera [19], where several hemocyte classes have been characterised. In coleopteran species, however, there is still limited information about the morphology and the specific functions of hemocytes [20].

**Figure 1:**
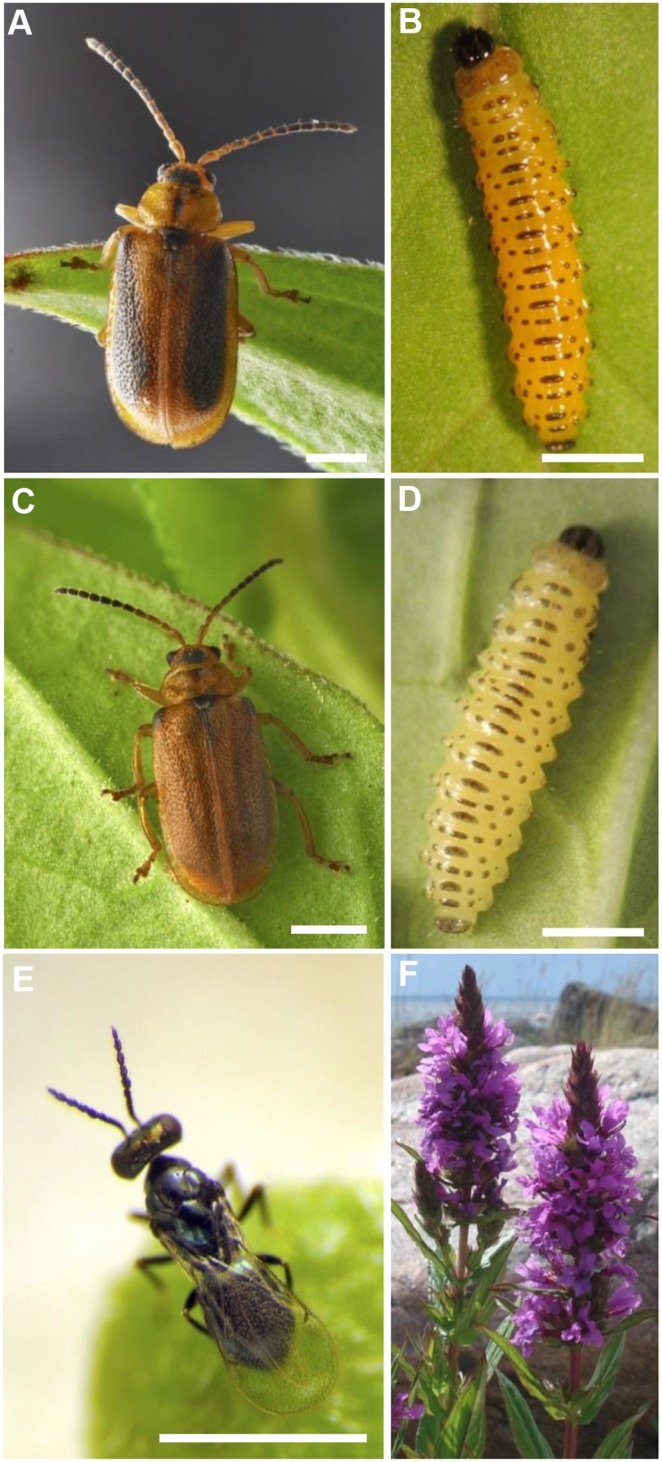
Study species. The two closely related beetle species (Coleoptera: Chrysomelidae); *Galerucella calmariensis* (A) adult and (B) larva and *Galerucella pusilla* (C) adult and (D) larva. (E) The parasitoid *Asecodes parviclava* (Hymenoptera: Eulophidae) and (F) the host plant *Lythrum salicaria* (Lythraceae). Scale bars: 1 mm.

Here we used two beetles, *Galerucella pusilla* Duftschmid and *G*. *calmariensis* L. (Coleoptera: Chrysomelidae) that share the same host plant, *Lythrum salicaria* L. (Lythraceae), and are attacked by the same parasitoid, *Asecodes parviclava* Thompson (Hymenoptera: Eulopidae) (Fig. 1). As the two *Galerucella* species are closely related with almost identical life cycles, they are well suited for comparative studies. The differentiation between the two *Galerucella* species is fairly recent, providing limited time for differences in immune responses to evolve [21]. Differences in the rate of parasitism between the two species have been observed [22], [23], indicating that *G. pusilla* experiences a lower parasitism rate than *G. calmariensis*. To gain a better understanding of the parasitism and the mechanisms behind the immune response for this system, we combined observations of interactions between *A. parviclava* and *Galerucella* spp with detailed studies of the hemocyte composition in the beetle larvae. We found considerable differences in the immune response between *G. pusilla* and *G. calmariensis*, potentially explaining differences in field parasitism rates. By studying larval hemolymph, we were able to distinguish several hemocyte types in the two species, including the cell types that are active in the encapsulation process of the immune response.

## Results

### Controlled parasitism experiments

In order to assess the parasitism level and the immune response we performed parasitism experiments with controlled numbers of *A. parviclava* parasitoids and lab-reared *Galerucella* larvae. In the experiments, most *G*. *calmariensis* (approximately 85%) showed clear signs of parasitoid attack prior to dissection, with one or more melanised spots in the cuticle (Fig. S1). At dissection, all *G. calmariensis* larvae proved to be successfully parasitised, containing one or more live parasitoid larvae (mean value 7.1 parasitoid larvae per beetle larva, range 1–20). In addition, the immune response in parasitised larvae was found to be quite ineffective; melanised eggs were found only in two *G. calmariensis* larvae (4%) and these larvae also contained live parasitoids. In *Galerucella pusilla* approximately 60% of the larvae showed cuticular melanisation at the wound site (Fig. S1). However, only 50% of the total number of larvae proved to be parasitised at dissection, indicating that the parasitoid attack was interrupted in some cases. In contrast to *G. calmariensis*, all parasitised *G. pusilla* showed a successful immune response, containing exclusively melanised eggs (mean value 4.5 eggs per larva, range 1–9) and no live parasitoid larvae.

### Cellular Composition

**Figure 2:**
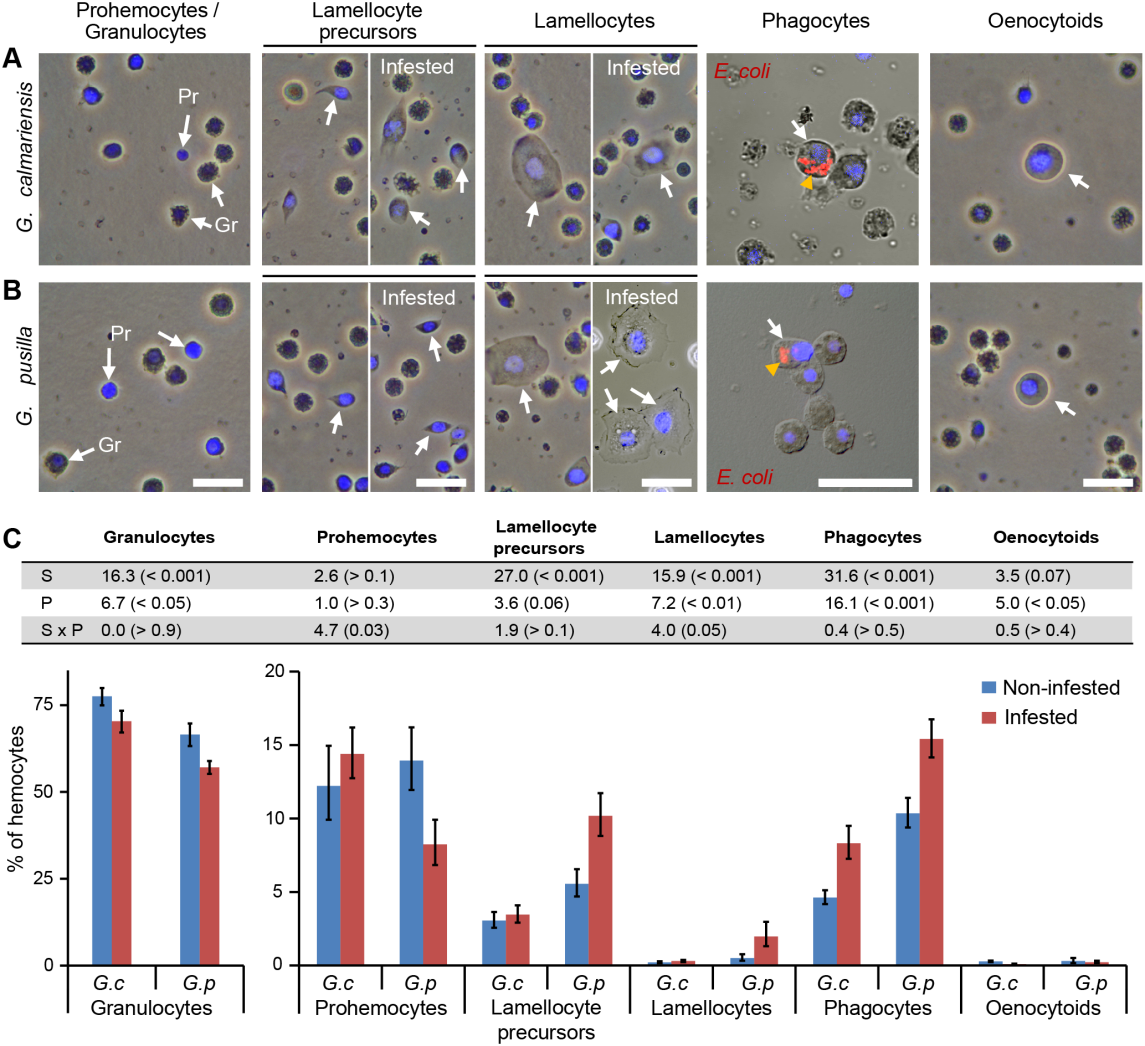
Morphology and differential counts of hemocytes in *Galerucella*. Morphology of hemocytes in (A) *G. calmariensis* and (B) *G. pusilla.* The white arrows indicate the cell types specified in the column headings. Both granulocytes (Gr) and prohemocytes (Pr) are shown in the first image. The orange arrowheads in the phagocyte images indicate the fluorescently-labelled bacteria inside the cells. Cell nuclei are stained with DAPI (blue). Scale bars: 20 µm. (C) Statistical table for separate ANOVAs, following the MANOVA, for six cell types between larvae of the two species (*G. calmariensis* and *G. pusilla*) and depending on parasitism by *A*. *parviclava*. F- and p-values (in brackets) are included and df_error_ = 56 (S: Species effect, P: Parasitism effect, S × P: Interactive effect). The column diagram shows differential hemocyte counts from laboratory-reared non-infested and infested *Galerucella* larvae (*G.c*: *G*. *calmariensis* [N_non-inf_ = 12, N_inf_ = 21], *G.p*: *G*. *pusilla* [N_non-inf_ = 11, N_inf_ = 16]). Error bars indicate standard error of the mean.

To unravel the reason for the difference in the parasitoid defense, the hemocyte composition was analysed and the ratios of different cell types in the two beetle species compared. Several hemocyte classes were distinguished morphologically in non-infested and infested individuals (Fig. 2A–B and Table S1): granulocytes, phagocytes, prohemocytes, oenocytoids, lamellocytes and lamellocyte precursors. The majority of hemocytes in non-infested individuals were granulocytes; round cells with rough surface, showing large vesicles in the cytoplasm; 77.5% in *G. calmariensis* and 66.5% in *G. pusilla* (Fig. 2A–C and Table S1). The granulocytes did not show any phagocytic activity according to our functional tests. In the circulation of non-infested individuals, 4.6% of the hemocytes in *G. calmariensis* and 10.4% in *G. pusilla* were found to be phagocytes, based on the injection of fluorescently labelled bacteria (Fig. 2A–C, Fig. S2 and Table S1). These cells can be morphologically distinguished from the granulocytes by the lack of granules. The phagocytes defined here are the functional equivalent of the plasmatocytes described in *Drosophila*, but in *Galerucella* the number of phagocytes is much lower (5–10%) than the number of plasmatocytes in *Drosophila* (99%) [18]. The prohemocytes; small cells with reduced cytoplasmic volume, constitute 12.2% of the circulating cells in non-infested *G. calmariensis* and 13.9% in *G. pusilla* (Fig. 2A–C and Table S1). Oenocytoids; large round cells with homogenous structure, (Fig. 2A–C and Table S1), were rarely observed in either species; 0.3% in non-infested *G. calmariensis* and 0.3% in *G. pusilla*. Lamellocyte precursors and lamellocytes were identified by phalloidin staining, their morphology and their reduced or absent phagocytic capacity (Fig. 2A–B, Table S1 and Fig. S2). In non-infested individuals, 3.1% of the circulating cells were found to be lamellocyte precursors in *G. calmariensis* and 5.6% in *G. pusilla* (Fig. 2C). Lamellocytes constitute 0.2% of the circulating cells in *G. calmariensis* and 0.5% in *G. pusilla* (Fig. 2C). In addition to the laboratory-reared larvae, the hemocyte composition in non-infested *G. calmariensis* and *G. pusilla* larvae collected from the field was investigated. When comparing non-infested lab-reared individuals with non-infested individuals from the field we found no difference in hemocyte composition in either species (Fig. S3).

### Clotting and hemocyte rupture

**Figure 3:**
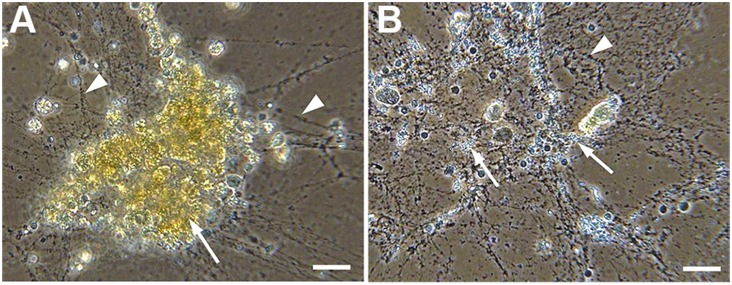
Clotting and hemocyte rupture in raw hemolymph of *Galerucella*. (A) Phase contrast image of a raw hemolymph sample from *G. calmariensis* containing large clusters of granulocytes (yellow, indicated by arrow) and clot matrix (dark fibres, indicated by arrowheads). The dissection was performed in paraformaldehyde to abolish the rupture of hemocytes. (B) Raw hemolymph sample 60 s after dissection of the larva. Note the absence of granulocytes. The content of the ruptured cells (indicated by arrows) and clot matrix (indicated by arrowheads) are visible. Scale bars: 20 µm.

**Figure 4:**
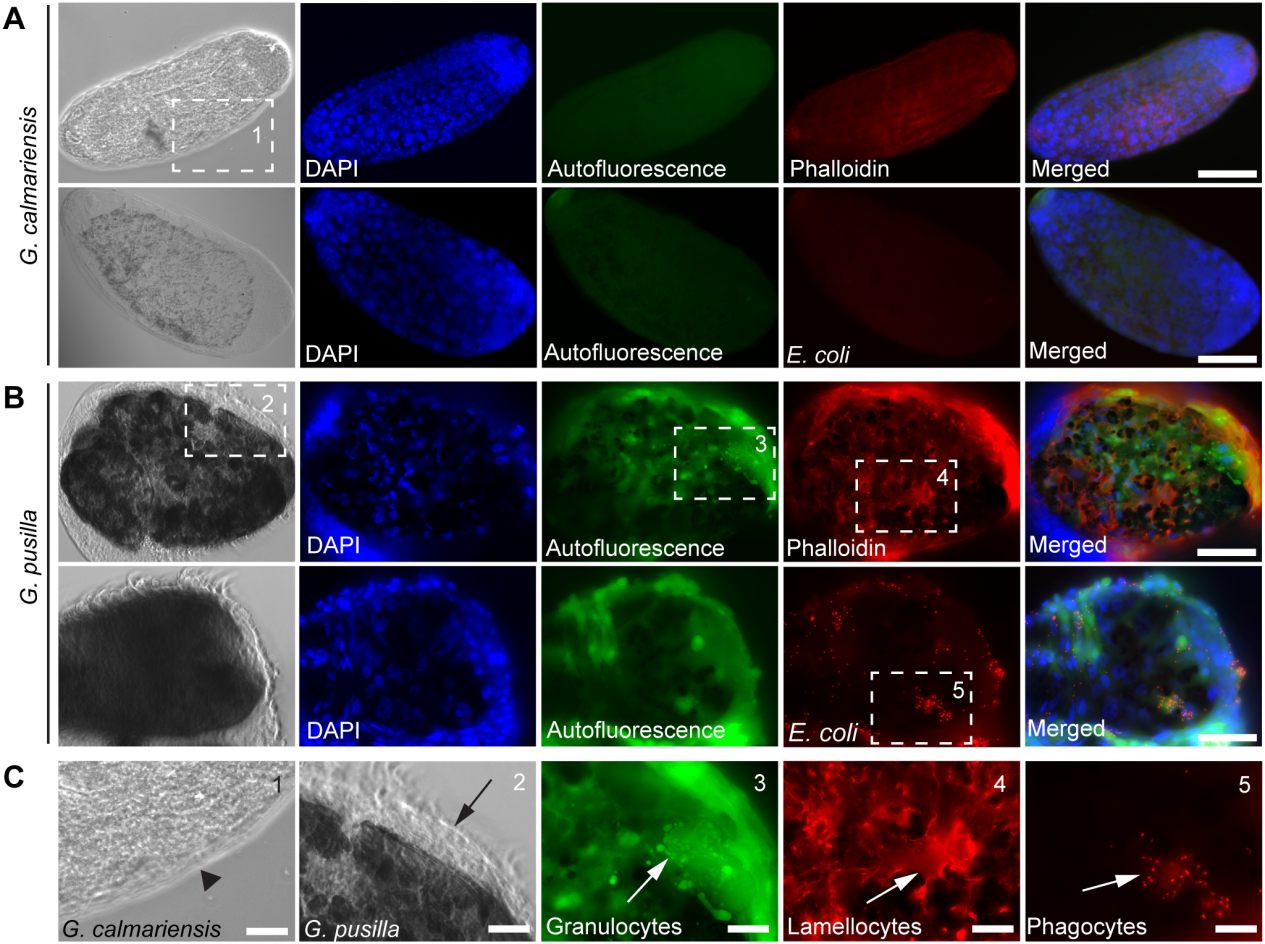
Immune response in *Galerucella* larvae towards the parasitoid *A. parviclava*. (A) Parasitoid eggs dissected from *G. calmariensis* and (B) encapsulated parasitoid eggs dissected from *G. pusilla.* The hemocytes are visualised by autofluorescence in the green channel, phalloidin staining (red) or engulfed fluorescent bacteria (red). Nuclei are stained with DAPI (blue). The first columns are the bright field images of parasitoid eggs. The blackening of the eggs from *G. pusilla* indicates melanisation. (C) Enlargements marked by dashed squares show the lack of cells on the parasitoid eggs from *G. calmariensis* (1), while several layers of cells are attached to the surface of eggs from *G. pusilla* (2). Granulocytes (3), lamellocytes (4) and phagocytes (5) are all visible on the surface of the encapsulated eggs from *G. pusilla*. Scale bars: a and b 25 µm, c 10 µm.

While establishing methods for hemocyte preparation we found that the hemolymph of *Galerucella* larvae coagulates within seconds. When analysing the raw hemolymph and staining for DNA, we observed a large population of clustered material in a network that includes the clot matrix (Fig. 3A). In addition, cell nuclei without cytoplasm surrounded by granules were observed. We suspected that rupture of hemocytes occurred at high speed; therefore the larvae were dissected in paraformaldehyde (PFA), to fix and slow down the processes. Large clusters of cells with similarity to granulocytes were detected in association with the clot matrix (Fig. 3B). To further characterise the behaviour of these cells, hanging drops of hemolymph [24] mixed with PBS were prepared and the cells monitored and recorded with 120 frames per second (Video S1). The rupture of the granulocytes takes approximately 50 ms and should thereby be a rapid way to deliver the content of the cell to the wound. We did not observe systemic rupture of granulocytes in the individuals injected with bacteria or infested by parasitoids, however, the granulocyte content was observed on the surface of the encapsulated parasitoid eggs (Fig. 4B–C) [25]. Additionally, when dissecting parasitoid eggs from *G. pusilla* large clusters of granulocytes surrounding the already melanised capsule were observed. Thus, we suggest that this cell type, similar to crystal cells in *Drosophila* [26], ruptures and delivers its cargo locally at the wound or infection site.

### Immune Response of *Galerucella*

In the parasitism experiments, only live parasitoid larvae were found in all *G. calmariensis* except two individuals, containing both live and encapsulated parasitoids. Conversely, in the infested *G. pusilla*, only encapsulated and melanised parasitoid eggs were found. In *G. calmariensis*, no hemocytes were seen attached to the parasitoid eggs in either bacteria-injected or phalloidin-stained samples, suggesting that capsule formation was inefficient already at a very early stage (Fig. 4A and C). In contrast, parasitoid eggs dissected from *G. pusilla* were covered by several layers of different hemocyte types (Fig. 4B–C and Fig. S2D–F). Confocal imaging of parasitoid eggs from both *Galerucella* species revealed that all three cell types; phagocytes, lamellocytes and granulocytes, contribute to the formation of the capsule. Phagocytes were detected with the help of fluorescent bacteria, lamellocytes by phalloidin staining and granulocytes by their autofluorescence in the green channel (Fig. 4). In order to investigate the difference between the species and the effect of infection on the hemocyte numbers we first performed a MANOVA with all cell types as response variables. When significant, post-hoc tests with separate ANOVAs were performed for each cell type. Since the cell counts were estimated as proportions, all values were logit-transformed prior to analysis. Residual plots suggested that this transformation normalised error distributions and homogenised variances. The MANOVA indicated that the cell type composition depended on species (F = 10.9, p0.001), infection status (F = 5.8, p0.001) and the interactions between these variables (F = 2.3, p0.05). The post-hoc tests suggested that the interactive effect was due to responses in lamellocytes and prohemocytes (the statistical table and backtransformed values on hemocyte ratios in the circulation of the two species are shown in Fig. 2C). Lamellocytes showed a larger difference between infested and non-infested larvae in *G*. *pusilla* than in *G*. *calmariensis.* The proportion of prohemocytes decreased following parasitism in *G*. *pusilla* but increased in *G*. *calmariensis*. The species effect was found in three other cell types in addition to lamellocytes; granulocytes, lamellocyte precursors and phagocytes. The number of granulocytes was lower in *G. pusilla* than in *G. calmariensis*, whereas the level of both lamellocyte precursors and phagocytes was higher in *G. pusilla* than in *G. calmariensis* (Fig. 2C). Parasitism was found to affect granulocytes, lamellocytes, phagocytes and oenocytoids. The number of granulocytes and oenocytoids decreased upon infection, whereas the level of both lamellocytes and phagocytes increased (Fig. 2C).

## Discussion

In this study we combined ecological and immunological aspects in order to understand interaction strengths in a natural host-parasitoid system, involving two closely related beetle species (*G. calmariensis* and *G. pusilla*) and their shared parasitoid (*A. parviclava*). Previous field observations suggested that *G. pusilla* experiences a much lower parasitism rate than *G. calmariensis*. Similarly, when comparing responses of the two beetles to the parasitoid in the laboratory, we found a striking difference in the successful parasitism rate on the two host species. This difference was traced to a corresponding difference in the level of successful immune response, with *G. pusilla* showing a much more potent immune defense towards the parasitoid.

When comparing the hemolymph of *G. pusilla* and *G. calmariensis*, we found that the ratios of granulocytes, lamellocytes, lamellocyte precursors and phagocytes differed between the two species. At least three cell types were found to be involved in the successful encapsulation of parasitoid eggs; lamellocytes, phagocytes and granulocytes. Lamellocytes and phagocytes were found to increase in numbers following parasitoid infection in both host species, although the constitutive levels of these cells were higher in *G. pusilla*, suggesting a better cellular immune competence in this species. Granulocytes decreased upon infection in both species, but the constitutive level of this cell type was higher in *G. calmariensis*. This suggests that granulocytes can be active not only in the capsule formation, but also in the clotting at the wound site, since the encapsulation process in *G. calmariensis* was rarely seen even though the number of granulocytes dropped. The level of oenocytoids also decreased after parasitism, suggesting an active role in the immune defence. So far, we have not detected oenocytoids in the encapsulation. However, these cells are known to be involved in the melanisation process in other species [27].

According to our observations, the ability of the host to increase the amount of lamellocytes seems crucial for the encapsulation process. This is in line with previous findings in *Drosophila melanogaster*, where lamellocytes are indispensable for proper capsule formation and for mounting an effective immune response against parasitoids [28]. Additionally, the phagocytic cells in *Drosophila melanogaster* contribute to the encapsulation, similar to what we observe in *Galerucella*. However, in non-infested *Drosophila melanogaster* 99% of the circulating cells are phagocytes ( =  plasmatocytes) [29], whereas the ratio of these cells in *Galerucella* is much lower. In *Drosophila* there is a subpopulation of phagocytes which develop into lamellocytes [30], showing plasticity of the immune response. We suggest that the lamellocyte precursors observed in *Galerucella* constitute a corresponding population of cells showing plasticity, as their phagocytic capacity is either low or absent. Additionally, there is a decrease in prohemocyte numbers in *G. pusilla* when infested, indicating that this species can differentiate new immune cells, whereas we found no evidence that this occurs in *G. calmariensis*.

The striking difference in immune competence between *G. pusilla* and *G. calmariensis* was unexpected, as the two species have almost identical life cycles, share the same host plant, and separated fairly recently [21]. Evolutionary, one may ask if the strong immune competence is an acquired trait in *G. pusilla* or a lost trait in *G. calmariensis*. As immune competence is dependent on the traits of both the parasitoid and the host, the direction of past evolution cannot be evaluated based on the present immune competence alone. Rather, a proper analysis necessitates information on genetic changes, to assess which regulatory changes have occurred in the host genome. Perhaps the strong immune response observed in *G. pusilla* indicates on-going evolution. The observation that virtually no parasitoid eggs survived in *G. pusilla* larvae might even raise the question whether *A*. *parviclava* can really be considered a true parasitoid on *G. pusilla*. In this study, only *A*. *parviclava* derived from *G. calmariensis* were used, which might suggest that the parasitoids have adapted to this host species, thereby explaining the low parasitism success in *G. pusilla*. On the other hand, parasitoids hatching from *G. pusilla* do not seem to be genetically differentiated from parasitoids hatching from *G. calmariensis* [21]. Moreover, the parasitism experiments have later been repeated several times with parasitoids hatching from *G. pusilla* with the same general results (L. Fors et al., in prep). Larvae of *G. calmariensis* show a very poor immune response regardless of the former host of the parasitoid, whereas *G. pusilla* has a strong immune response also when attacked by parasitoids hatching from its own species (roughly 25% of the *G. pusilla* larvae manage to encapsulate parasitoid eggs of *A*. *parviclava* hatching from other *G. pusilla* hosts). However, even though the parasitism rates on *G. pusilla* are generally low also in the field, we have observed high parasitism rates on *G. pusilla* in some localities. Whether this geographic variation is due to increased virulence in *A*. *parviclava* or reduced immune competence in *G. pusilla* is presently unknown. Studies in other systems suggest that geographic variation in both immune competence and virulence is common [31], and may occur both because of genetic and environmental differences among sites. The immune competence in the host often depends strongly on both microclimate and host feeding conditions [32].

The strong immune competence in *G*. *pusilla* may come at a cost. In other species, it has been shown that life history trade-offs between growth, reproduction and immune competence are common [3], [33]. Such trade-offs have not been investigated within the *Galerucella* beetles, but we do know that *G*. *calmariensis* has a 15% higher larval growth rate than *G*. *pusilla* in the field and also reaches a larger adult size [34]. A shorter development time for the larvae means a shorter exposure to parasitoids, and thereby a lower risk for parasitism. According to our observations in the laboratory, the parasitoids need to attack the beetle larvae at a quite early stage in order for successful parasitism to occur, regardless of the immune defense in the beetles. If the beetle larva is close to pupation when attacked by the parasitoid, there will not be sufficient time for the parasitoid larvae to develop.

In conclusion, our studies reveal that the main reason for the different parasitism rates on the two closely related *Galerucella* species is based on differences in their immune competence. There was a close correspondence between the hemocyte composition in the two species and their ability to mount an effective immune response against the parasitoid *A*. *parviclava*. The prominent differences in immune competence between the two beetles strongly suggest that field studies on host-parasitoid interactions would gain from a better understanding of the underlying immunological responses.

## Materials and Methods

### Ethic statement

No endangered or protected species were involved in this study. All sites used for field collection are located on privately owned land (for site coordinates see below). According to Swedish law no permission is needed from private land owners for field sampling.

### Study species

*Galerucella pusilla* Duftschmid and *G*. *calmariensis* L. (Coleoptera: Chrysomelidae) both use *Lythrum salicaria* L. (Lythraceae) exclusively as host plant, for feeding and oviposition. The beetles have similar life cycles; they over-winter as adults and emerge during spring. Eggs are deposited directly onto the leaves or stem of *L*. *salicaria* and hatch after a few weeks. The larvae pupate in the ground 3–4 weeks later and the next generation of adults emerge 2–3 weeks after pupation. In Sweden *G. pusilla* occurs from the south up until Sundsvall [N 62°, E17°], whereas *G. calmariensis* is common also further north. The adult morphology is similar between the species, but larvae can be easily distinguished based on colour (Fig. 1). *G. calmariensis* usually have bright yellow coloured larvae, while the larvae of *G. pusilla* have a pale white-yellow tone [34].

*Asecodes parviclava* Thompson (Hymenoptera: Eulophidae) is a small (<1 mm) parasitic wasp known to attack *G. pusilla* and *G. calmariensis* in the larval stage, laying one or more eggs inside the larva [35]. The parasitoid larvae develop inside their host, which makes the beetle larva unable to pupate. Rather, it turns into a mummified black shell from which the adult parasitoids subsequently hatch, usually during the next summer [34]. The mean number of parasitoids hatching from a parasitised larva is 4.7 [23].

For our experiments, adult beetles were collected in the field in mid-May and held in the laboratory for mating and oviposition. Two field sites in the south-east of Sweden were used for collection; one in the county of Uppland [N59.852°, E18.107°] and one in the county of Gävleborg [N60.873°, E17.326°]. At these sites both beetle species occur. *G. calmariensis* was also collected in two sites in the north of Sweden [N63.773°, E20.624°; N63.790°, E20.625°]. Approximately 50 males and 50 females of each species were collected at respective field site. The beetles from the mixed localities were distinguished by looking at the spurs on the meso- and metatibiae of the adults [36]. To verify species identity the colour of each larva was checked prior to the experiments. The eggs were kept in the laboratory until hatching, thus there was no risk of larvae being parasitised prior to the tests. The larvae used for experiments and hemocyte samples were in the second instar and of approximately the same size. In addition to laboratory-reared larvae, larvae were also collected in the field at the sites mentioned above and included as controls (Fig. S3). The parasitoids used for the parasitism experiments were all females, collected from previous season’s parasitised pupae. Only parasitoids hatching from *G. calmariensis* from the north of Sweden were used, since parasitoid abundance was much higher in this area. To distinguish between the sexes, the parasitoids were anesthetised with carbon dioxide and separated based on overall size and the morphology of the gaster. Males are generally smaller than females with a slender gaster, whereas the female gaster has more rounded sides and an ovipositor which is visible in ventral view (Christer Hansson, pers. comm.).

### Controlled parasitism experiments

To investigate the level of parasitism, 10 laboratory-reared larvae of each species were put in a 200 ml transparent plastic container together with 5 *A. parviclava* females. The parasitoids were removed after 24 h, to ensure sufficient time for all beetle larvae to be parasitised. The larvae were thereafter monitored in a stereo microscope to detect melanised wound sites in the cuticle, indicating parasitoid attack. They were kept for an additional 96 h before dissection, to make sure that parasitoid larvae were large enough to be easily detected during dissection, and to provide sufficient time for possible encapsulation processes to be completed. All beetle larvae were provided with fresh leaves of *L*. *salicaria* each day of the experiment. At dissection the percentage of successfully parasitised beetle larvae (containing live parasitoid larvae) and the percentage of larvae showing a successful immune response (containing melanised eggs) were determined. In total, 50 *G. calmariensis* larvae (20 respectively 10 originating from the southern localities, and 10 from each of the two northern localities) and 30 *G. pusilla* larvae (20 respectively 10 originating from the southern localities) were used in the parasitism tests.

### Bleeding procedure and preparation of hemocytes

Hemocyte samples were prepared from all larvae used in the parasitism experiments. Additional hemocyte samples, serving as controls, were prepared from non-infested larvae reared in the laboratory, and from non-infested larvae collected in the field. The larvae were carefully rinsed in Phosphate buffered saline (PBS) prior to dissection. To avoid hemolymph clotting and melanisation each larva was submerged in a well containing 300 µl PBS mixed with a small amount of phenylthiourea (PTU), and dissected by holding the head firmly and tearing the cuticle on the dorsal side with the tips of the forceps. The hemolymph was homogenized with a pipette tip before preparing the samples. In earlier trials the cells of *Galerucella* were found to be extremely prone to rupture when in contact with air, which is the reason for keeping the larvae in PBS at dissection.

Three hemocyte samples were prepared from each individual larva. For each sample, a 30 µl drop of the mixture (hemolymph + PBS) was placed on a multi-spot microscope slide (SM-011, Hendley, Loughton, U.K.) and left to adhere for 45 min in a humid chamber at room temperature. After adhesion excessive hemolymph was gently removed with a pipette and the remaining cell monolayer was fixed with 4% PFA + PBS for 10 min. After fixation cells were washed twice with PBS for 5 min. To reveal the nuclei the cells were treated with blue-fluorescent nucleic acid stain DAPI (Sigma-Aldrich, 1∶1000 dilution in PBS) for 10 min. After DNA-staining the cells were washed twice with PBS for 5 min and mounted in Fluoromount-G (SouthernBiotech) mounting media.

### Identification of hemocyte types

To investigate the presence of phagocytic cells, a suspension of Texas-Red labeled, heat killed *E. coli* bacteria was injected into live larvae of both beetle species. The larvae were either exposed to parasitoid attack prior to the injection, or non-parasitised larvae only injected with bacteria. Both field-collected and laboratory-reared larvae were included. After 30 min the larvae were bled and hemocyte samples were prepared as before. In order to identify the cell types involved in the encapsulation process, paired samples of hemocytes and encapsulated parasitoid eggs dissected from infested *Galerucella* were permeabilised (Triton-X 100, 0.05%) for 5 min and incubated with Rhodamine Phalloidin (Molecular Probes/Life Technologies, 1∶50) and DAPI diluted in PBS.

All samples were studied in a Zeiss Axioplan2, phase contrast, epifluorescent microscope connected to a Hamamatsu camera with Axio Vision 4.6. Nine random images per individual were taken and used for differential hemocyte counts. On average 850 hemocytes per individual were counted and grouped according to morphology (Table S1). Parasitoid eggs and hemocyte samples from the bacterial injections were investigated by epifluorescent and confocal microscopy.

## Supporting Information

Figure S1 **Cuticular melanisation in** ***Galerucella*** **larvae.** (A) Larva of *G. calmariensis* showing cuticular melanisation at the wound site after parasitoid attack. (B) Larva of *G. pusilla* with a melanised wound in the cuticle after bacteria injection.
(TIF)

Figure S2 **Functional characterisation of hemocytes in** ***Galerucella*** **.** (A) and (B) show hemocyte samples from *G*. *calmariensis* larvae after *in vivo* phagocytosis assay. Arrows indicate lamellocyte in (A) and lamellocyte precursor in (B). Orange arrowheads indicate phagocytes. (C) Phalloidin staining of hemocytes from parasitoid infested *G*. *pusilla* larvae. (D–F) High magnification image of the cellular multilayer formed around the parasitoid egg in *G*. *pusilla*. Hemocytes are stained with rhodamin-phalloidin (red) and nuclei with DAPI (blue). Scale bars: 20 µm.
(TIF)

Figure S3 **Differential hemocyte counts of** ***Galerucella*** **from the field.** Hemocyte counts of non-infested *G. calmariensis* and *G. pusilla* larvae collected in the field compared to non-infested, laboratory-reared larvae (G.c: *G*. *calmariensis* [N_lab_ = 12, N_field_ = 9], *G.p*: *G*. *pusilla* [N_lab_ = 11, N_field_ = 11]). Error bars indicate standard error of the mean.
(TIF)

Table S1 **Identification key for** ***Galerucella*** **hemocytes.**
(PDF)

Table S2 **Raw data for hemocyte counts in** ***G*** **.** ***calmariensis*** **and** ***G*** **.** ***pusilla*** **.** The data from each experimental group (non-infested lab-reared larvae, infested lab-reared larvae and non-infested field collected larvae) of the two species are shown in separate excel sheets named accordingly. The tables include image file names, dates for the experiments, sample identification data, geographic origin of the beetles, the raw hemocyte counts subdivided into hemocyte classes and the calculated percentages. On average 850 hemocytes from 3–9 images were counted per individual.
(XLSX)

Video S1 **Rupture of granulocytes in** ***Galerucella*** **.** Hanging drops of hemolymph mixed with PBS prepared from *Galerucella* larvae, and recorded with 120 frames per second. The rupture of one granulocyte is shown, taking approximately 50 ms.
(MP4)

## References

[1] Vilcinskas A (2013) Evolutionary plasticity of insect immunity. J Insect Physiol 59: 123–129. 22985862 10.1016/j.jinsphys.2012.08.018

[2] Kraaijeveld AR, Godfray HCJ (1999) Geographic patterns in the evolution of resistance and virulence in *Drosophila* and its parasitoids. Am Nat 153: S61–S74. 29578778 10.1086/303212

[3] Schulenburg H, Kurtz J, Moret Y, Siva-Jothy MT (2009) Ecological immunology. Philos Trans R Soc Lond B Biol Sci 364: 3–14. 18926970 10.1098/rstb.2008.0249 PMC2666701

[4] Kraaijeveld AR, Van Alphen JJM, Godfray HCJ (1998) The coevolution of host resistance and parasitoid virulence. Parasitology 116: S29–S45. 9695108 10.1017/s0031182000084924

[5] Lemaitre B, Hoffmann J (2007) The host defense of *Drosophila melanogaster* . Annu Rev Immunol 25: 697–743. 17201680 10.1146/annurev.immunol.25.022106.141615

[6] Chapman RF (1969) The Insects: Structure and Function. New York: Cambridge University Press. 959 p.

[7] Kounatidis I, Ligoxygakis P (2012) *Drosophila* as a model system to unravel the layers of innate immunity to infection. Open Biol 2. Available: http://rsob.royalsocietypublishing.org/content/2/5/120075. 10.1098/rsob.120075 PMC3376734 22724070

[8] Dushay MS (2009) Insect hemolymph clotting. Cell Mol Life Sci 66: 2643–2650. 19418022 10.1007/s00018-009-0036-0 PMC11115950

[9] Giglio A, Battistella S, Talarico FF, Brandmayr TZ, Giulianini PG (2008) Circulating hemocytes from larvae and adults of *Carabus* (*Chaetocarabus*) *lefebvrei* Dejean 1826 (Coleoptera, Carabidae): Cell types and their role in phagocytosis after in vivo artificial non-self-challenge. Micron 39: 552–558. 17825571 10.1016/j.micron.2007.07.004

[10] Lavine MD, Strand MR (2002) Insect hemocytes and their role in immunity. Insect Biochem Mol Biol 32: 1295–1309. 12225920 10.1016/s0965-1748(02)00092-9

[11] Theopold U, Schmidt O, Söderhäll K, Dushay MS (2004) Coagulation in arthropods: defence, wound closure and healing. Trends Immunol 25: 289–294. 15145318 10.1016/j.it.2004.03.004

[12] Salt G (1967) Cellular defense mechanisms in insects. Fed Proc 26: 1671–1674. 6075905

[13] Wertheim B, Kraaijeveld AR, Schuster E, Blanc E, Hopkins M, et al (2005) Genome-wide gene expression in response to parasitoid attack in *Drosophila* . Genome Biol 6: R94 Available: http://genomebiology.com/2005/6/11/r94. 16277749 10.1186/gb-2005-6-11-r94 PMC1297650

[14] Pham LN, Schneider DS (2008) Evidence for specificity and memory in the insect innate immune response. In: Beckage NE, editor. Insect Immunology: Academic Press. 97–127.

[15] Mikkola K, Rantala MJ (2010) Immune defence, a possible nonvisual selective factor behind the industrial melanism of moths (Lepidoptera). Biol J Linn Soc Lond 99: 831–838.

[16] Ojala K, Julkunen-Tiito R, Lindström L, Mappes J (2005) Diet affects the immune defence and life-history traits of an Arctiid moth *Parasemia plantaginis* . Evol Ecol Res 7: 1153–1170.

[17] Rizki RM, Rizki TM (1990) Encapsulation of parasitoid eggs in phenoloxidase-deficient mutants of *Drosophila melanogaster* . J Insect Physiol 36: 523–529.

[18] Kurucz E, Vaczi B, Markus R, Laurinyecz B, Vilmos P, et al (2007) Definition of *Drosophila* hemocyte subsets by cell-type specific antigens. Acta Biol Hung 58: S95–S111. 10.1556/ABiol.58.2007.Suppl.8 18297797

[19] Jiang H, Vilcinskas A, Kanost MR (2010) Immunity in lepidopteran insects. Adv Exp Med Biol 708: 181–204. 21528699 10.1007/978-1-4419-8059-5_10 PMC9284565

[20] Manachini B, Arizza V, Parrinello D, Parrinello N (2011) Hemocytes of *Rhynchophorus ferrugineus* (Olivier) (Coleoptera: Curculionidae) and their response to *Saccharomyces cerevisiae* and *Bacillus thuringiensis* . J Invertebr Pathol 106: 360–365. 21147119 10.1016/j.jip.2010.12.006

[21] Hambäck PA, Weingartner E, Ericson L, Fors L, Cassel-Lundhagen A, et al (2013) Bayesian species delimitation reveals generalist and specialist parasitic wasps on *Galerucella* beetles (Chrysomelidae): sorting by herbivore or plant host. BMC Evol Biol 13: 92 Available: http://www.biomedcentral.com/1471-2148/13/92. 23622105 10.1186/1471-2148-13-92 PMC3662573

[22] Stenberg JA, Heijari J, Holopainen JK, Ericson L (2007) Presence of *Lythrum salicaria* enhances the bodyguard effects of the parasitoid *Asecodes mento* for *Filipendula ulmaria* . Oikos 116: 482–490.

[23] Hambäck PA, Stenberg JA, Ericson L (2006) Asymmetric indirect interactions mediated by a shared parasitoid: connecting species traits and local distribution patterns for two chrysomelid beetles. Oecologia 148: 475–481. 16502320 10.1007/s00442-006-0387-2

[24] Bidla G, Lindgren M, Theopold U, Dushay MS (2005) Hemolymph coagulation and phenoloxidase in *Drosophila* larvae. Dev Comp Immunol 29: 669–679. 15854679 10.1016/j.dci.2004.11.007

[25] Ratcliffe NA, Rowley AF (1979) Roles of hemocytes in defense against biological agents. In: Gupta AP, editor. Insect hemocytes. New York: Cambridge University Press 331–414.

[26] Bidla G, Dushay MS, Theopold U (2007) Crystal cell rupture after injury in *Drosophila* requires the JNK pathway, small GTPases and the TNF homolog Eiger. J Cell Sci 120: 1209–1215. 17356067 10.1242/jcs.03420

[27] Shrestha S, Kim Y (2008) Eicosanoids mediate prophenoloxidase release from oenocytoids in the beet armyworm *Spodoptera exigua* . Insect Biochem Mol Biol 38: 99–112. 18070669 10.1016/j.ibmb.2007.09.013

[28] Rizki TM, Rizki RM (1992) Lamellocyte differentiation in *Drosophila* larvae parasitized by *Leptopilina* . Dev Comp Immunol 16: 103–110. 1499832 10.1016/0145-305x(92)90011-z

[29] Kurucz E, Markus R, Zsamboki J, Folkl-Medzihradszky K, Darula Z, et al (2007) Nimrod, a putative phagocytosis receptor with EGF repeats in *Drosophila* plasmatocytes. Curr Biol 17: 649–654. 17363253 10.1016/j.cub.2007.02.041

[30] Honti V, Csordas G, Markus R, Kurucz E, Jankovics F, et al (2010) Cell lineage tracing reveals the plasticity of the hemocyte lineages and of the hematopoietic compartments in *Drosophila melanogaster* . Mol Immunol 47: 1997–2004. 20483458 10.1016/j.molimm.2010.04.017

[31] Carton Y, Poirie M, Nappi AJ (2008) Insect immune resistance to parasitoids. Insect Sci 15: 67–87.

[32] Lazzaro BP, Little TJ (2009) Immunity in a variable world. Philos Trans R Soc Lond B Biol Sci 364: 15–26. 18926975 10.1098/rstb.2008.0141 PMC2666692

[33] Gwynn DM, Callaghan A, Gorham J, Walters KFA, Fellowes MDE (2005) Resistance is costly: trade-offs between immunity, fecundity and survival in the pea aphid. Proc Biol Sci 272: 1803–1808. 16096092 10.1098/rspb.2005.3089 PMC1559873

[34] Hambäck P (2004) Why purple loosestrife in sweet gale shrubs are less attacked by herbivorous beetles? Entomol Tidskr 125: 93–102.

[35] Hansson C, Hambäck PA (2013) Three cryptic species in *Asecodes* (Forster) (Hymenoptera, Eulophidae) parasitizing larvae of *Galerucella* spp. (Coleoptera, Chrysomelidae), including a new species. J Hymenopt Res 30: 51–64.

[36] Cossé AA (2004) Presence of tibial spurs as a male sexual character for *Galerucella calmariensis* (Coleoptera : Chrysomelidae). J Entomol Sci 39: 281–283.

