# Supplementary figures and images for: Differences in Cellular Immune Competence Explain Parasitoid Resistance for Two Coleopteran Species
Source: PLoS One. 2014 Sep 26;9(9):e108795. doi: 10.1371/journal.pone.0108795 (PMC4178244; doi:10.1371/journal.pone.0108795)

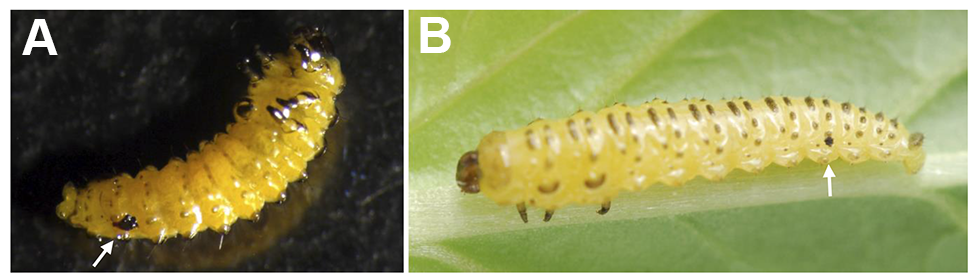

Supplement: Figure S1 — Cuticular melanisation in Galerucella larvae. (A) Larva of G. calmariensis showing cuticular melanisation at the wound site after parasitoid attack. (B) Larva of G. pusilla with a melanised wound in the cuticle after bacteria injection. (TIF) [file pone.0108795.s001.tif]

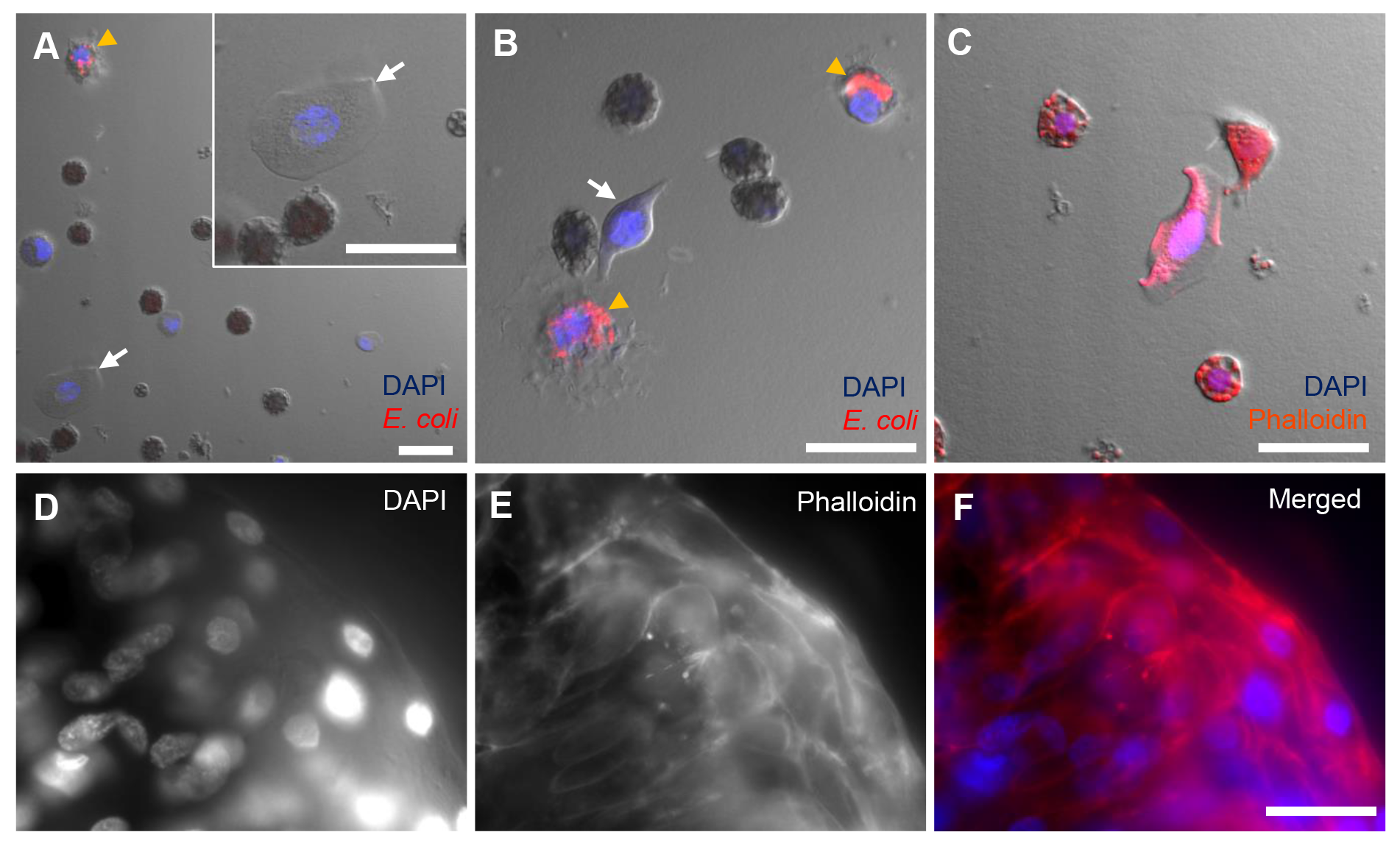

Supplement: Figure S2 — Functional characterisation of hemocytes in Galerucella . (A) and (B) show hemocyte samples from G. calmariensis larvae after in vivo phagocytosis assay. Arrows indicate lamellocyte in (A) and lamellocyte precursor in (B). Orange arrowheads indicate phagocytes. (C) Phalloidin staining of hemocytes from parasitoid infested G. pusilla larvae. (D–F) High magnification image of the cellular multilayer formed around the parasitoid egg in G. pusilla. Hemocytes are stained with rhodamin-phalloidin (red) and nuclei with DAPI (blue). Scale bars: 20 µm. (TIF) [file pone.0108795.s002.tif]

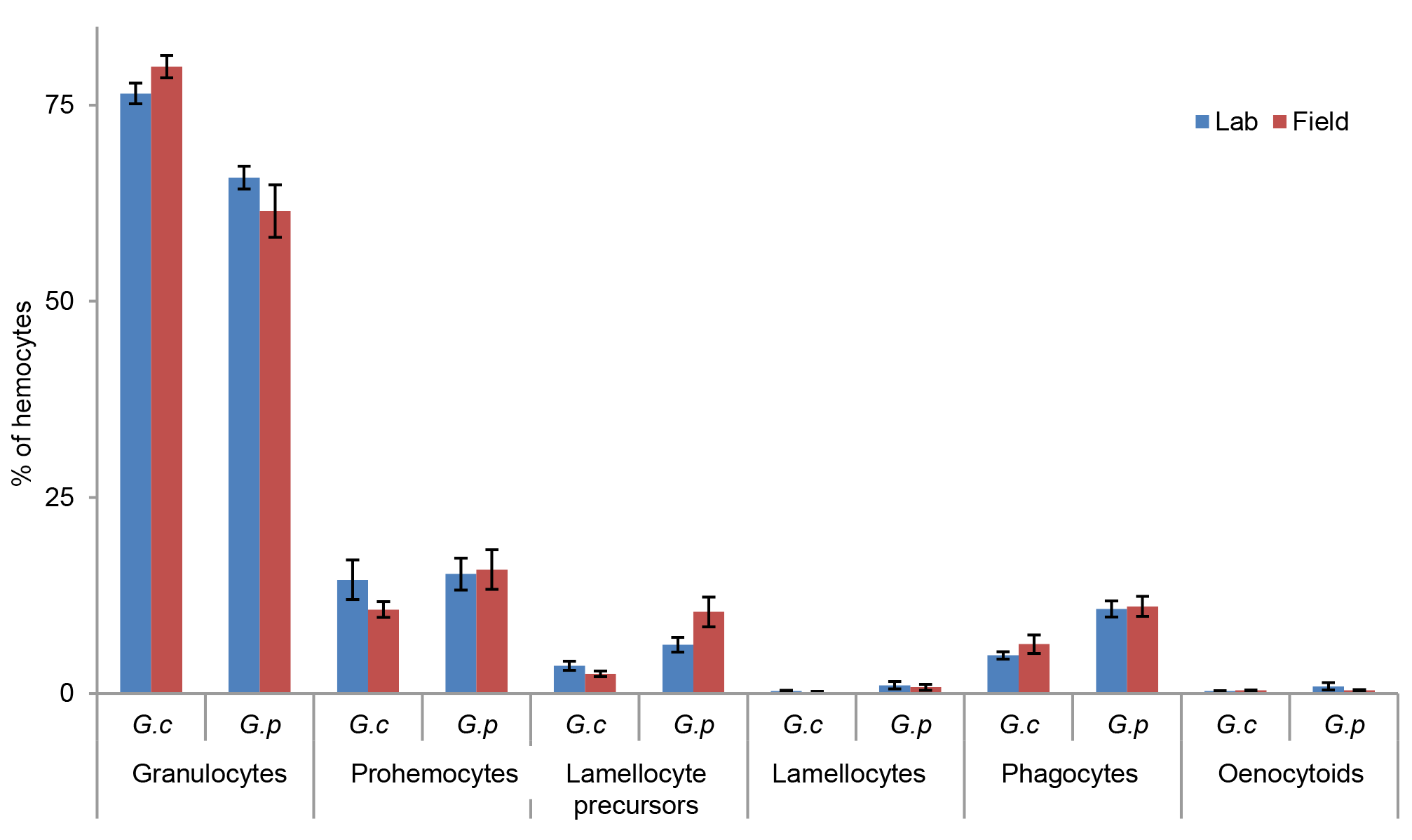

Supplement: Figure S3 — Differential hemocyte counts of Galerucella from the field. Hemocyte counts of non-infested G. calmariensis and G. pusilla larvae collected in the field compared to non-infested, laboratory-reared larvae (G.c: G. calmariensis [Nlab = 12, Nfield = 9], G.p: G. pusilla [Nlab = 11, Nfield = 11]). Error bars indicate standard error of the mean. (TIF) [file pone.0108795.s003.tif]
