# Supplementary material for: Differences in Cellular Immune Competence Explain Parasitoid Resistance for Two Coleopteran Species
Source: PLoS One. 2014 Sep 26;9(9):e108795. doi: 10.1371/journal.pone.0108795 (PMC4178244; doi:10.1371/journal.pone.0108795)
Supplement: Table S1 — Identification key for Galerucella hemocytes. (PDF) [file pone.0108795.s004.pdf]

Hemocyte class	Cell shape	Nucleus	Cytoplasm
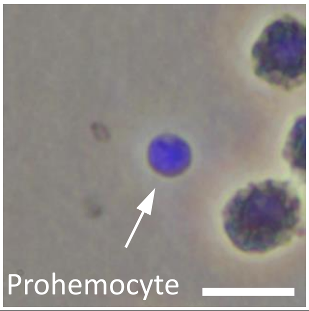	Round	Strong DAPI staining	Homogenous, narrow rim, dark grey in phase contrast, no colour in differential interference contrast (DIC)
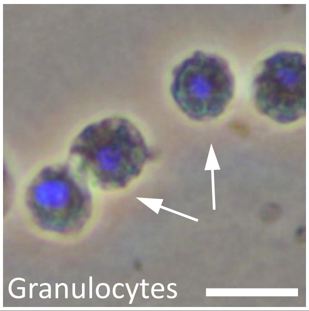	Round, rough surface	Small, weaker DAPI staining than the other cells	Granular morphology, yellowish tint in brightfield or DIC, grey in phase contrast
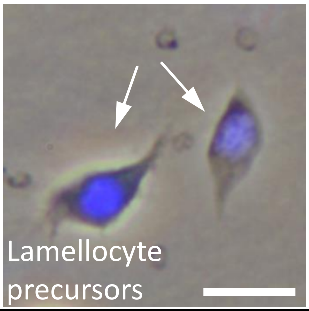	Spindle	Strong DAPI staining	Homogenous, dark grey in phase contrast, no colour in DIC
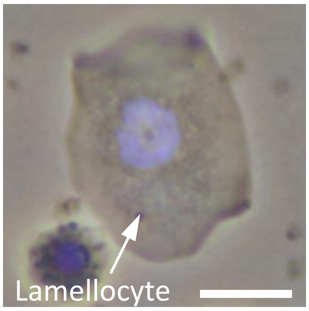	Large, flat, rectangular or triangular	Weak DAPI staining	Fine grey structure around the nucleus, light grey in phase contrast
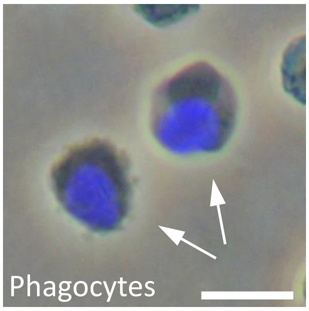	Round, slightly irregular	Strong DAPI staining	Homogenous, dark grey in phase contrast, no color in DIC (if fluorescent bacteria are injected see fig 1)
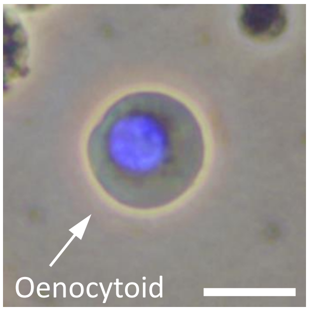	Round, smooth surface	Strong DAPI staining	Homogenous, glossy yellow-brown aspect, with strong phase ring in phase contrast, no colour in DIC, but the cell appears to be the thickest of all

Scale bars: 10 µm
